# Supplementary material for: Ta3N5 Nanobelt-Loaded Ru Nanoparticle Hybrids’ Electrocatalysis for Hydrogen Evolution in Alkaline Media
Source: Molecules. 2023 Jan 21;28(3):1100. doi: 10.3390/molecules28031100 (PMC9919797; doi:10.3390/molecules28031100)
Supplement: Supplementary file 1 [file molecules-28-01100-s001.zip › molecules-2152778-supplementary.pdf]

Supplementary materials

Ta_3N_5 Nanobelt-Loaded Ru Nanoparticle Hybrids' Electrocatalysis for Hydrogen Evolution in Alkaline Media

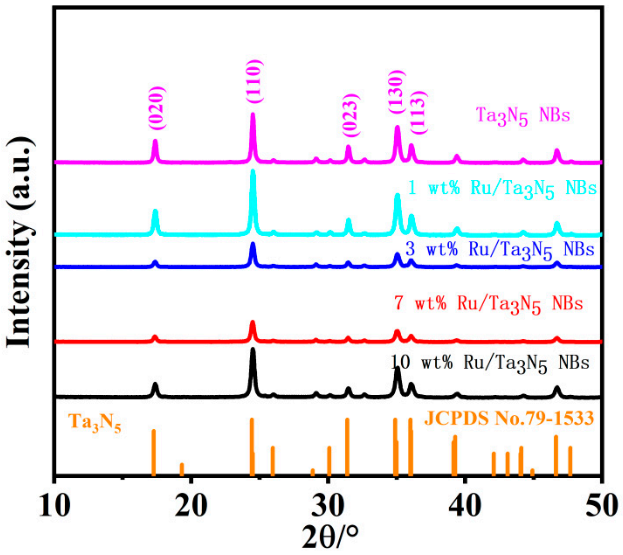

Figure S1. XRD patterns of Ta_3N_5 NBs, and 1, 3, 7, and 10 wt% Ru/Ta_3N_5 NBs.

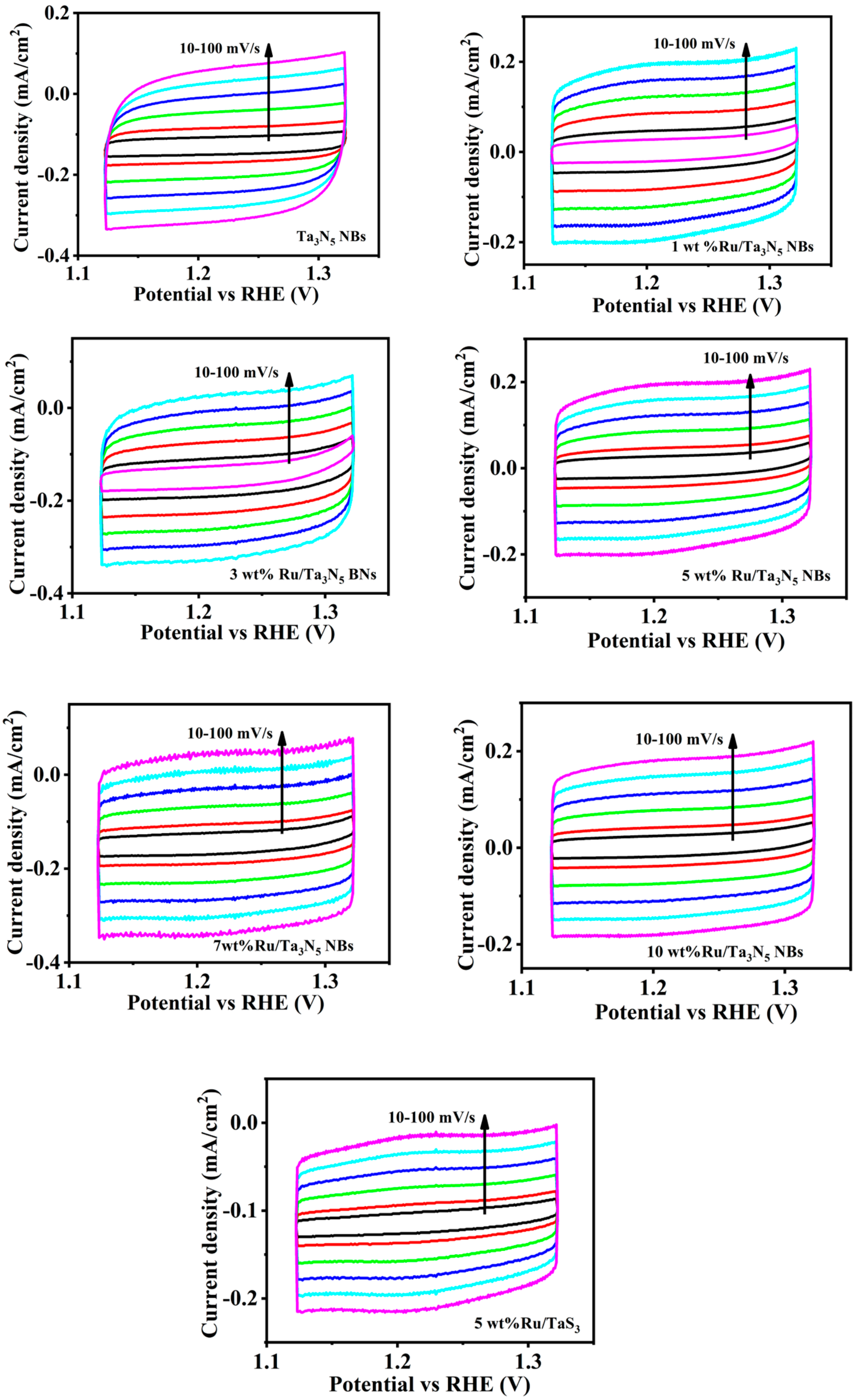

Figure S2. The CV curves at potentials from 1.1 to 1.35 V in a nitrogen-saturated 1 M KOH (vs. RHE) of Ta_3N_5 NBs, 1, 3, 5, 7 and 10 wt% Ru/Ta_3N_5 NBs, and 5 wt% Ru/TaS_3 NBs.

Detail DFT calculations: The spin polarization DFT calculations were carried out by the Dmol[3] module in Materials Studio 8.0 package and generalized gradient approximation with Perdew–Becke–Ernzerhof (PBE) was used for the exchange–correlation functional. The double numerical plus polarization (DNP) basis set were adopted, while an

accurate DFT semi-core pseudopots (DSPP) was employed for the metal atoms. All of the models are calculated in periodically boxes with a vacuum slab of 15 Å to separate the interaction between periodic images. The optimized bulk structures were used to construct surface slab models. A (2 × 2) Ru (100) slab model of 7 layers with supercell of 5.345× 8.465 ×21.944 Å (α=β=γ=90°) used for pure Ru, with the bottom 5 layers of Ru atoms fixed to mimic bulk structure. A (2 × 2) Ta_3N_5 (001) slab model of 10 layers with supercell of 11.039× 7.807 ×24.117 Å (α=β=90°, γ= 69.29 °) was used for pure Ta_3N_5, with the bottom 8 layers of atoms fixed to mimic bulk structure. Ru cluster was constructed on (2 × 2) Ta_3N_5 (001) slab model of 10 layers formed supercell with 11.039× 7.807 ×24.117 Å (α=β=90°, γ= 69.29 °). All the transition state configurations were confirmed through the frequency analysis. The energy, gradient and displacement convergence criteria were set as 1×10^{-5} Ha, 2×10^{-3} Å and 5×10^{-3}Å, respectively. The Gibbs free energy of each elementary step was calculated as

$$\Delta G = \Delta E + \Delta ZPE - T\Delta S,$$

where ΔE is the reaction energy calculated using the spin polarization DFT method. ΔZPE and ΔS are the changes in zero-point energies and entropy during the reaction, respectively. Particularly, as the vibrational entropy of H* in the adsorbed state is small, the entropy of adsorption of 1/2 H_2 is $\Delta S_H \approx -0.5S_{0,H2}$, where $S_{0,H2}$ is the entropy of H_2 in the gas phase at the standard conditions. The adsorption energy of hydrogen was defined as E_{slab-H} - E_{slab} -1/2E_{H2}.
